# Supplementary material for: Immune profiling of age and adjuvant-specific activation of human blood mononuclear cells in vitro
Source: Commun Biol. 2024 Jun 8;7:709. doi: 10.1038/s42003-024-06390-4 (PMC11162429; doi:10.1038/s42003-024-06390-4)
Supplement: Supplementary file 2 — Supplementary Information [file 42003_2024_6390_MOESM2_ESM.pdf]

SUPPORTING INFORMATION

Immune profiling of age and adjuvant-specific activation of human blood mononuclear cells *in vitro*

Simone S. Schüller[1,2,ω,†], Soumik Barman[1,2,†], Raul Mendez-Giraldez[3], Dheeraj Soni[1,2,#], John Daley[4], Lindsey R. Baden[2,5], Ofer Levy[1,2,6,ϕ,**], David J. Dowling[1,2,ϕ,**]

[1]*Precision Vaccines Program*, Boston Children's Hospital, Boston, MA, USA, [2]Harvard Medical School, Boston, Massachusetts, USA, [3]Beckman Coulter Life Sciences, CA, USA, [4]Dana Farber CyTOF Core Facility, Dana-Farber Cancer Institute, Boston, MA, USA, [5]Department of Medicine, Brigham and Women's Hospital, Boston, MA, USA, [6]Broad Institute of MIT & Harvard, Cambridge, MA, USA

[ω]Current Address: Neonatal Directorate, Child and Adolescent Health Service, Perth, Australia

[#]Current Address: Sanofi, Cambridge, MA, USA

[†]These authors contributed equally: Simone S. Schüller and Soumik Barman

[ϕ]These authors jointly supervised this work: Ofer Levy and David J. Dowling

[ϕ] Correspondence: O.L. [ofer.levy@childrens.harvard.edu] and D.J.D. [david.dowling@childrens.harvard.edu]

INDEX

Supplementary Tables		Page
Table 1	Panel and reagents for mass cytometry assay.	S-3
Table 2	Immune cell populations and phenotypic definition. Phenotypic definition was adopted from (25, 27, 93).	S-4
Table 3	Biological samples, reagents, and algorithms for mass cytometry.	S-5

Supplementary Figures		Page
Figure 1	Quality control of the cytometry files using PeacoQC algorithm by the Cytobank platform.	S-6
Figure 2	Gating strategy for MNCs, T cells and NK cells.	S-7
Figure 3	Immunophenotyping the baseline of cell lineages involved in the innate and adaptive arm of each study participant.	S-8
Figure 4	Activation profile of co-stimulatory molecule CD86 on mDCs, pDCs and monocytes after PRRa stimulation.	S-9
Figure 5	Activation profile of co-stimulatory molecule CD40 on mDCs, pDCs and monocytes after PRRa stimulation.	S-10
Figure 6	TLR7/8a (R848) has greater IFNγ inducing efficacy than other PRRa in human cord BMCs.	S-11
Figure 7	TLR7/8a (R848) has greater IFNγ inducing efficacy than other PRRa in human adult BMCs.	S-12
Figure 8	TLR7/8a (R848) has greater IFNγ inducing efficacy than other PRRa in human elder BMCs.	S-13
Figure 9	Intracellular cytokines profile of MNCs after PRRa stimulation for 18h.	S-14
Figure 10	Dissecting TLR7/8a-specific IFNγ producing cell subsets in each study participant.	S-15
Figure 11	Tracking the IFNγ producing cellular lineages.	S-16
Figure 12	Clustering analysis identifies 7 different cellular populations of IFNγ producing cells with age-specific differences for γδ T cells in adults and $CD4^+$ T cell in later life.	S-17
Figure 13	Characterization of nodes (16, 22 & 30) from the SPADE bubble of $IFN\gamma^+$ γδ T cell compartment after TLR7/8a (R848) stimulation.	S-18

Supplementary Table 1. Panel and reagents for mass cytometry assay. Intracellular cytokines were highlighted by light gold color.

Index	Target	Clone	Metal	Vendor	Identifier
1	CD45	HI30	89Y	Standard BioTools	3089003B
2	IFNα	LT27:295	115In	Miltenyi	N/A*
3	CD235a/b	HIR2	141Pr	Standard BioTools	3141001B
4	CD19	HIB19	142Nd	Standard BioTools	3142001B
5	CD4	RPA-T4	145Nd	Standard BioTools	3145001B
6	CD8a	RPA-T8	146Nd	Standard BioTools	3146001B
7	CD20	2H7	147Sm	Standard BioTools	3147001B
8	CD16	3G8	148Nd	Standard BioTools	3148004B
9	CD25	2A3	149Sm	Standard BioTools	3149010B
10	MIP1β	D21-1351	150Nd	Standard BioTools	3150004B
11	CD123	6H6	151Eu	Standard BioTools	3151001B
12	TNFα	Mab11	152Sm	Standard BioTools	3152002B
13	CD80	2D10.4	155Gd	Miltenyi	N/A*
14	CD86	IT2.2	156Gd	Standard BioTools	3156008B
15	CD33	WM53	158Gd	Standard BioTools	3158001B
16	CD11c	Bu15	159Tb	Standard BioTools	3159001B
17	CD14	M5E2	160Gd	Standard BioTools	3160001B
18	IL-23p19	23dcdp	161Dy	Standard BioTools	3161010B
19	CD27	L128	162Dy	Standard BioTools	3162009B
20	CD34	581	163Dy	Standard BioTools	3163014B
21	IL-17A	N49-653	164Dy	Standard BioTools	3164002B
22	CD40	5C3	165Ho	Standard BioTools	3165005B
23	IL-10	JES3-9D7	166Er	Standard BioTools	3166008B
24	CCR7	G043H7	167Er	Standard BioTools	3167009A
25	IFNγ	B27	168Er	Standard BioTools	3168005B
26	CD45RA	HI100	169Tm	Standard BioTools	3169008B
27	CD3	UCHT1	170Er	Standard BioTools	3170001B
28	CD66a	CD66a-B1.1	171Yb	Standard BioTools	3171004B
29	CD38	HIT2	172Yb	Standard BioTools	3172007B
30	TCRγδ	11F2	173Yb	BioLegend	N/A*
31	HLA-DR	L243	174Yb	Standard BioTools	3174001B
32	CD71	OKT-9	175Lu	Standard BioTools	3175011B
33	CD56	NCAM16.2	176Yb	Standard BioTools	3176008B
34	Cell-ID Intercalator	-	191Ir	Standard BioTools	201192B
35	Cell-ID Intercalator	-	193Ir	Standard BioTools	201192B
36	Viability	-	195Pt	Standard BioTools	201195
37	CD11b	ICRF44	209Bi	Standard BioTools	3209003B

* Custom conjugated antibodies were indicated by N/A.

Supplementary Table 2. Immune cell populations and phenotypic definition.

Index	Populations	Phenotypic definition
1	Mononuclear cells (MNC)	$CD45^{+}$ $CD66a^{-}$
2	Plasmablasts	MNC^{+} $CD3^{-}$ $CD19^{+}$ $CD16^{-}$ $CD56^{-}$ $CD14^{-}$ $CD20^{-}$ $CD38^{+}$ $CD27^{+}$
3	B cells	MNC^{+} $CD3^{-}$ $CD19^{+}$ $CD16^{-}$ $CD56^{-}$ $CD14^{-}$ $CD20^{+}$
4	B Memory	B $Cells^{+}$ $CD27^{+}$ HLA-$DR^{dim, +}$
5	B Naive	B $Cells^{+}$ CD27- HLA-$DR^{dim, +}$
6	γδ T cells	MNC^{+} $CD3^{+}$ $CD19^{-}$ $TCRgd^{dim, +}$ $CD14^{-}$ $CD4^{-}$ $CD8^{-}$
7	Double positive (DP) T cells	MNC^{+} $CD3^{+}$ $CD19^{-}$ $TCRgd^{-}$ $CD11c^{-}$ $CD14^{-}$ $CD4^{+}$ $CD8^{+}$
8	Double negative (DN) T cells	MNC^{+} $CD3^{+}$ $CD19^{-}$ $TCRgd^{-}$ $CD11c^{-}$ $CD14^{-}$ $CD4^{-}$ $CD8^{-}$
9	NKT cells	MNC^{+} $CD3^{+}$ $CD19^{-}$ $TCRgd^{-}$ $CD11c^{-}$ $CD14^{-}$ $CD4^{-}$ $CD56^{+}$
10	CD8 T cells	MNC^{+} $CD3^{+}$ $CD19^{-}$ $TCRgd^{-}$ $CD11c^{-}$ $CD14^{-}$ $CD4^{-}$ $CD8^{+}$
11	CD8 Effector Memory cells	CD8 T $cells^{+}$ $CCR7^{-}$ $CD27^{+}$
12	CD8 Terminal Effector cells	CD8 T $cells^{+}$ $CCR7^{-}$ $CD27^{-}$
13	CD8 Central Memory cells	CD8 T $cells^{+}$ $CD45RA^{-}$ $CCR7^{+}$ $CD27^{+}$
14	CD8 Naïve cells	CD8 T $cells^{+}$ $CD45RA^{+}$ $CCR7^{+}$ $CD27^{+}$
15	CD4 T cells	MNC^{+} $CD3^{+}$ $CD19^{-}$ $TCRgd^{-}$ $CD11c^{-}$ $CD14^{-}$ $CD4^{+}$ $CD8^{-}$
16	Th1 cells	CD4 T $cells^{+}$ $IFN\gamma^{+}$ / TNF^{+}
17	CD4 Effector Memory cells	CD4 T $cells^{+}$ $CD45RA^{-}$ $CCR7^{-}$ $CD27^{+}$
18	CD4 Terminal Effector cells	CD4 T $cells^{+}$ $CD45RA^{-}$ $CCR7^{-}$ $CD27^{-}$
19	CD4 Central Memory cells	CD4 T $cells^{+}$ $CD45RA^{-}$ $CCR7^{+}$ $CD27^{+}$
20	CD4 Naïve cells	CD4 T $cells^{+}$ $CD45RA^{+}$ $CCR7^{+}$ $CD27^{+}$
21	T_{reg} cells	CD4 T $cells^{+}$ $CD25^{+}$
22	NK Cells	MNC^{+} $CD3^{-}$ $CD19^{-}$ HLA-$DR^{dim, -}$ $CD56^{dim, +}$ $CD123^{-}$ $CD14^{-}$ $CD45RA^{+}$
23	Monocytes	MNC^{+} $CD3^{-}$ $CD19^{-}$ HLA-DR^{+} $CD56^{-}$ $CD11c^{+}$
24	Classical monocytes	Monocytes+ $CD14^{+}$ $CD38^{+}$
25	Non-classical monocytes	Monocytes+ $CD14^{-}$ $CD38^{-}$
26	Transitional monocytes	Monocytes+ $CD14^{dim}$ $CD38^{dim}$
27	Myeloid dendritic cells (mDCs)	MNC^{+} $CD3^{-}$ $CD19^{-}$ $CD14^{-}$ $CD20^{-}$ HLA-DR^{+} $CD123^{-}$ $CD11c^{+}$ $CD38^{dim, +}$ $CD16^{-}$
28	Plasmacytoid DCs (pDCs)	MNC^{+} $CD3^{-}$ $CD19^{-}$ $CD14^{-}$ $CD20^{-}$ HLA-DR^{+} $CD123^{+}$ $CD11c^{-}$
29	Basophils	MNC^{+} $CD3^{-}$ $CD19^{-}$ HLA-DR^{-} $CD56^{-}$ $CD123^{+}$
30	Eosinophils	$CD45^{+}$ $CD66a^{+}$ $CD3^{-}$ $CD19^{-}$ HLA-DR^{-} $CD16^{-}$
31	Neutrophils	$CD45^{+}$ $CD66a^{+}$ $CD16^{+}$ $CD3^{-}$ HLA-DR^{-}

Supplementary Table 3. Biological samples, reagents and algorithms for mass cytometry assay.

Reagent or Resource	Source	Identifier
Biological samples		
BMCs from newborn cord (38-40 weeks), healthy adult (22-63 years) and elder (65-85 years) participants were collected before the COVID-19 pandemic	The use of human blood samples for this study was approved by the Institutional Review Board of the BCH. See "**Methods**"	See "**Supplementary Data 2**"
Chemicals, TLR agonists		
Ficoll-Paque PREMIUM	Cytiva	17544203
Synthetic Monophosphoryl Lipid A (MPLA) (TLR4)	InvivoGen	tlrl-mpls
Resiquimod (R848) (TLR7/8)	InvivoGen	tlrl-r848-5
ODN 2216 (Class A CpG oligonucleotide) (TLR9)	InvivoGen	tlrl-2216-5
Alhydrogel® adjuvant 2% (Alum)	InvivoGen	vac-alu-250
Human TruStain FcX™	BioLegend	422302
Cell-ID 20-Plex Pd Barcoding Kit	Standard BioTools	201060
Maxpar® Barcode Perm Buffer	Standard BioTools	201057
Maxpar® Water	Standard BioTools	201069
Maxpar® PBS	Standard BioTools	201058
Maxpar® Cell Staining Buffer	Standard BioTools	201068
Maxpar® Fix I Buffer	Standard BioTools	201065
Maxpar® Perm-S Buffer	Standard BioTools	201066
Maxpar® Cell Acquisition Solution	Standard BioTools	201240
EQ Four Element Calibration Beads	Standard BioTools	201078
GolgiPlug (containing Brefeldin A)	BD Biosciences	555029
GolgiStop (containing Monensin)	BD Biosciences	554724
Software and Algorithms		
Cytobank	Cytobank	https://premium.cytobank.org/cytobank/
PeacoQC	Cytobank	https://premium.cytobank.org/cytobank/
tSNE-CUDA	Cytobank	https://premium.cytobank.org/cytobank/
SPADE	Cytobank	https://premium.cytobank.org/cytobank/
Prism 10 (Version 10.2) for macOS	GraphPad Software	https://www.graphpad.com
EndNote 21	Clarivate	https://endnote.com
Microsoft 365	Microsoft	https://www.microsoft.com/en-us/microsoft-365
Deposited Data		
FCS files for CyTOF	Cytobank	https://premium.cytobank.org/cytobank/projects/3668

A

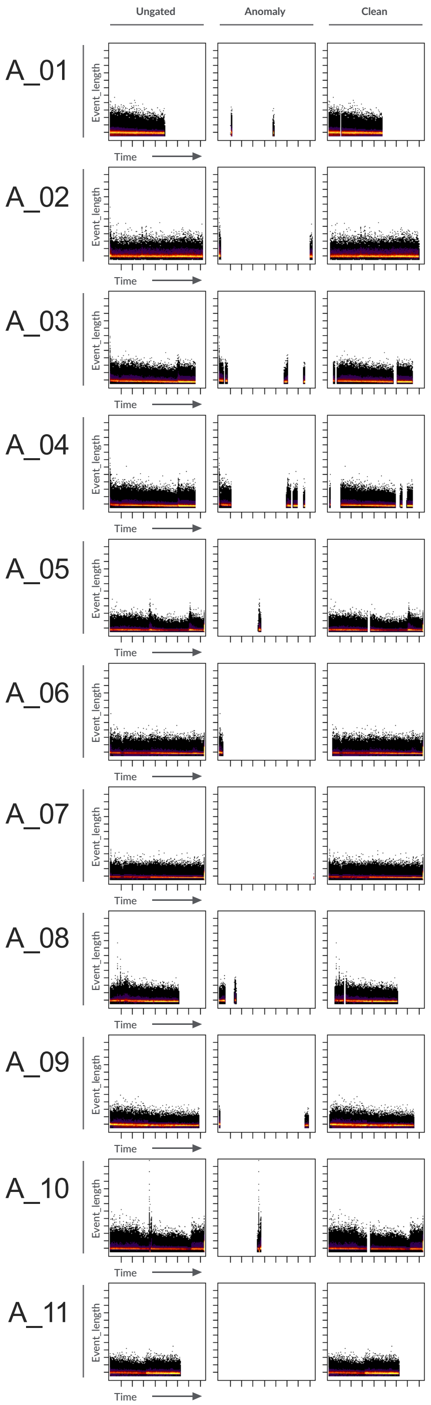

B

	Ungated	Anomaly	Clean
A_01	100	3.445	96.555
A_02	100	5.901	94.099
A_03	100	14.307	85.693
A_04	100	27.056	72.944
A_05	100	3.785	96.215
A_06	100	3.693	96.307
A_07	100	0.00161	99.998
A_08	100	11.082	88.918
A_09	100	5.371	94.629
A_10	100	3.708	96.292
A_11	100	0	100

Supplementary Figure 1: Quality control of the cytometry files using PeacoQC algorithm by the Cytobank platform. (A) Representative plot (from adult cohort after R848 stim) of ungated, anomaly and clean raw normalized events after PeacoQC run. (B) Statistics, which is directly exported from the Cytobank platform showing the percent of population varying by columns of ungated events. Clean population was selected for downstream manual gating according to Supplementary Table 2.

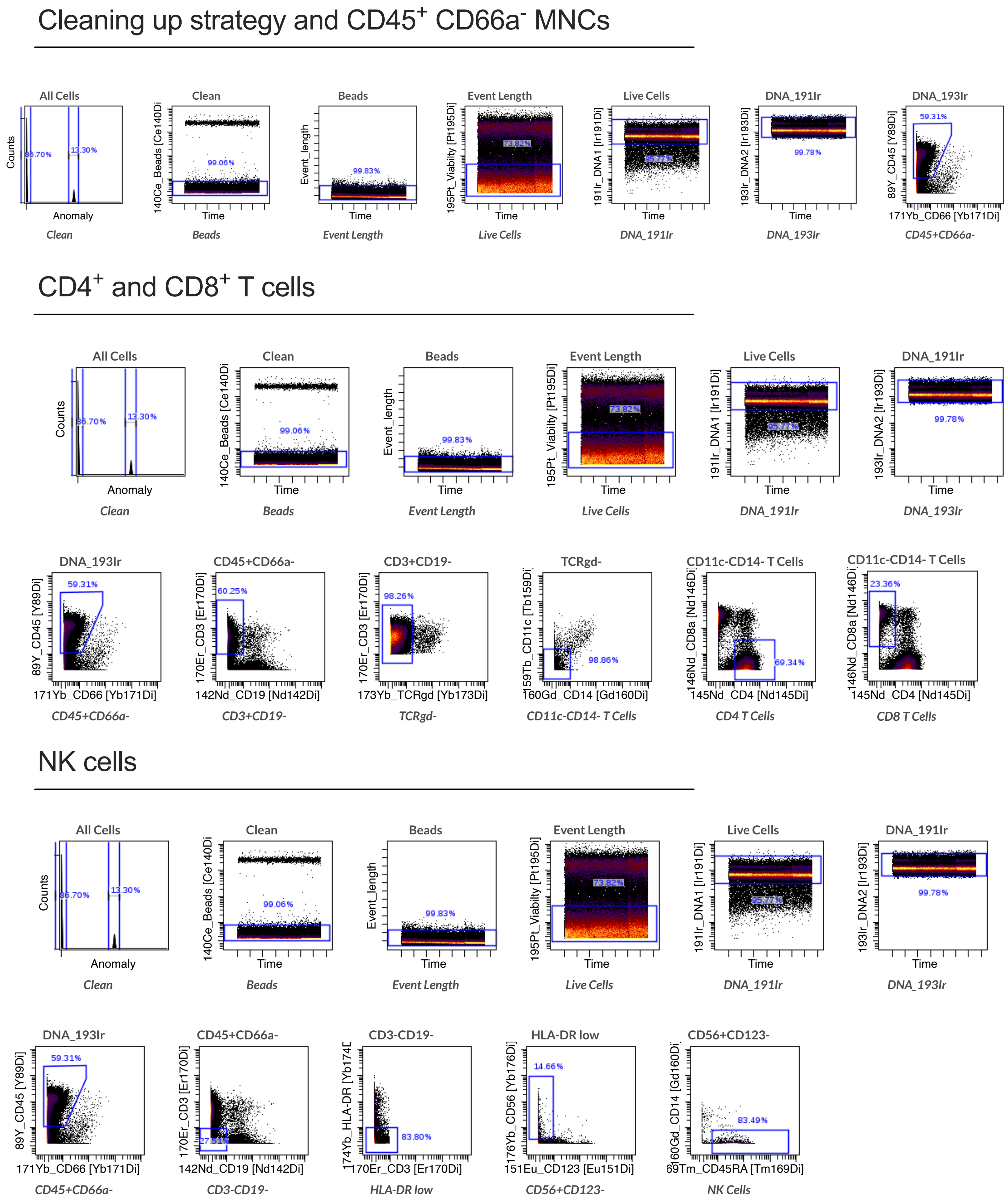

Supplementary Figure 2: Gating strategy for MNCs, T cells and NK cells. A representative gating strategy after cleaning up the cytometry files using PeacoQC algorithm followed by phenotyping of CD45+ CD66a- cells (MNCs), CD4+ or CD8+ T cells and NK cells. Manual gating was accomplished by the Cytobank platform.

A

Newborn

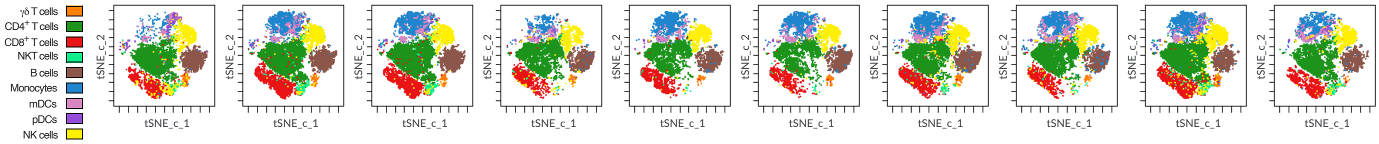

γδ T cells	2.901	0.397	0.404	0.511	0.439	0.943	0.577	0.287	0.529	0.0886
CD4$^+$ T cells	14.327	27.39	24.936	40.51	33.067	21.874	20.5	30.49	38.38	17.556
CD8$^+$ T cells	6.243	10.78	10.344	13.564	11.861	10.294	10.653	6.039	15.627	5.566
NKT cells	1.164	1.143	1.036	0.07	0.15	0.119	0.194	0.111	1.816	1.193
B cells	10.527	8.837	7.934	9.31	13.8	14.021	12.314	12.177	3.954	10.193
Monocytes	11.989	7.4	10.811	1.844	6.374	9.669	10.47	12.299	9.733	12.531
mDCs	3.404	0.809	0.63	0.666	0.966	0.847	1.357	1.519	1.211	2.813
pDCs	0.66	0.0386	0.0429	0.0329	0.0729	0.101	0.0714	0.06	0.116	0.321
NK cells	2.601	4.076	3.713	2.793	5.749	5.894	2.914	6.759	4.804	10.73

B

Adult

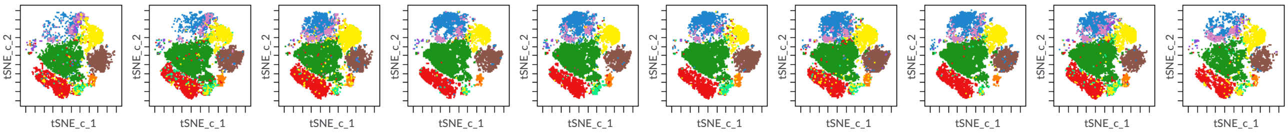

γδ T cells	0.86	0.824	4.12	0.916	2.073	4.153	3.096	0.367	1.59	0.341
CD4$^+$ T cells	13.567	32.826	12.241	32.393	29.389	28.85	21.149	42.383	33.77	12.136
CD8$^+$ T cells	7.979	23.414	7.801	11.196	10.994	9.661	11.247	8.921	20.989	8.193
NKT cells	0.653	2.804	0.274	0.963	0.343	2.613	1.221	0.156	4.427	1.234
B cells	7.516	13.313	5.856	7.301	9.584	19.627	13.164	4.411	5.553	7.91
Monocytes	8.767	3.426	4.73	8.113	14.099	8.273	10.739	7.5	9.42	5.906
mDCs	2.254	0.471	0.936	0.749	1.819	0.767	1.111	0.861	1.35	2.094
pDCs	0.203	0.0757	0.0443	0.134	0.12	0.1	0.0771	0.0457	0.129	0.187
NK cells	3.964	3.174	10.82	5.336	4.27	3.043	6.874	4.93	6.204	9.51

C

Elder

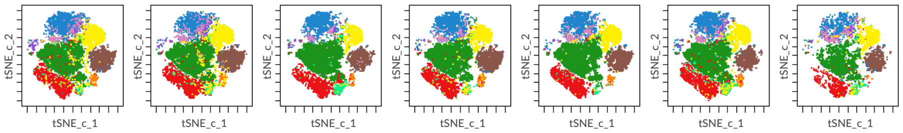

γδ T cells	0.657	0.53	0.306	1.333	0.354	0.681	0.0371
CD4$^+$ T cells	23.909	33.801	39.704	34.226	38.993	30.09	10.663
CD8$^+$ T cells	6.831	8.2	12.287	7.364	8.427	15.786	2.886
NKT cells	0.341	0.874	3.654	0.606	0.137	2.856	0.837
B cells	4.909	3.781	4.239	11.019	6.117	3.977	8.94
Monocytes	8.729	9.406	8.491	8.459	13.201	10.117	12.551
mDCs	0.754	1.157	0.7	0.359	1.933	0.806	3.081
pDCs	0.116	0.194	0.0929	0.0414	0.17	0.117	0.3
NK cells	3.937	3.473	1.909	2.399	7.74	6.381	7.151

Supplementary Figure 3: Immunophenotyping the baseline of cell lineages involved in the innate and adaptive arm of each study participant. DR analysis using tSNE-CUDA of CD45$^+$ CD66a$^-$ MNCs from each individual (n=27) without any stimulation. Data from 3 participants were excluded for downstream analysis as they did not fit the threshold criteria of 70K events/ individuals as described in Figure 1. Percent of population varying by overlaid (selected cell populations) of CD45$^+$ CD66a$^-$ MNCs were shown in tables from (A) newborn, (B) adult and (C) elder cohorts. Data were directly exported from the Cytobank platform.

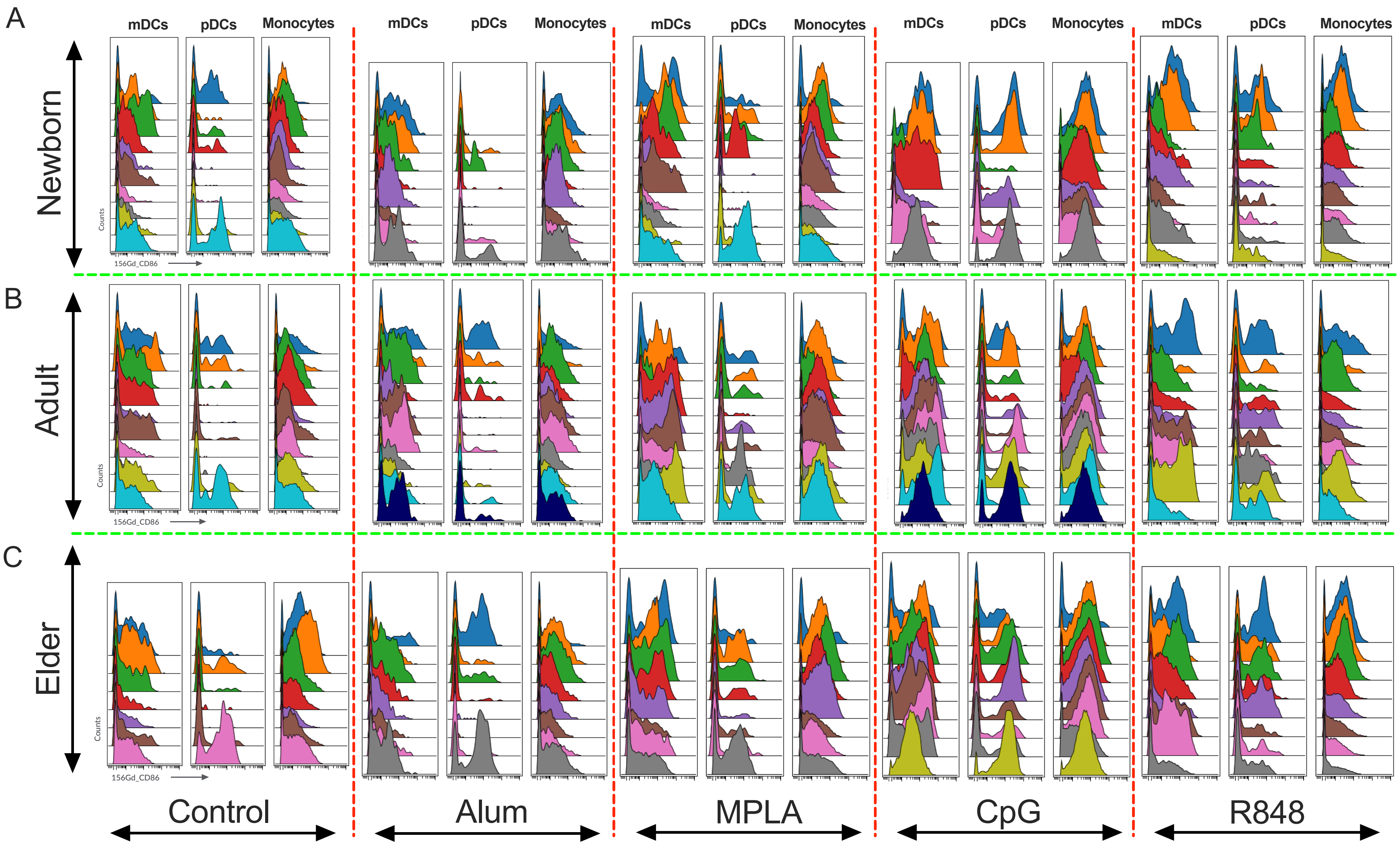

Supplementary Figure 4: Activation profile of co-stimulatory molecule CD86 on mDCs, pDCs and monocytes after PRRa stimulation. Median metal intensity (Med MI) of CD86 expression on mDCs, pDCs and monocytes in the (A) newborn, (B) adult and (C) elder cohorts. Color (randomly assigned but in a numerical order) in histogram indicates each participant's (n=27) CD86 activation profile upon PRRa stimulation. Non-stimulated BMCs (control) or stimulated with alum (10µg/ml), MPLA (100ng/ml), CpG (5µM) and R848 (5µM) for 18h. Green dotted lines separate each cohort whereas red dotted lines separate each stimulant. Data were directly exported from the Cytobank platform.

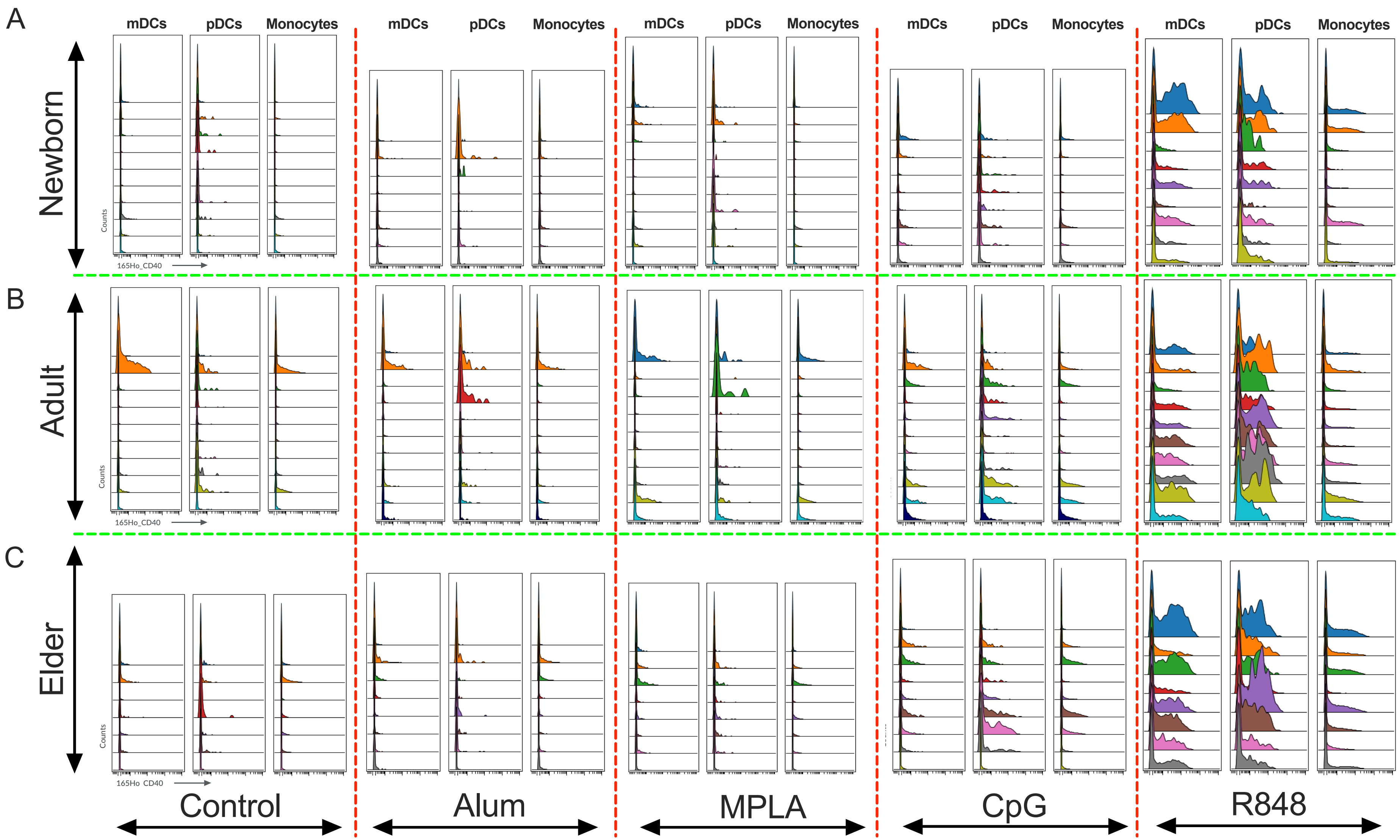

Supplementary Figure 5: Activation profile of co-stimulatory molecule CD40 on mDCs, pDCs and monocytes after PRRa stimulation. Median metal intensity (Med MI) of CD40 expression on mDCs, PDCs and monocytes in the (A) newborn, (B) adult and (C) elder cohorts. Color in histogram indicates each participant's CD40 activation profile upon PRRa stimulation. Non-stimulated BMCs (control) or stimulated with alum (10µg/ml), MPLA (100ng/ml), CpG (5µM) and R848 (5µM) for 18h. Green dotted lines separate each cohort whereas red dotted lines separate each stimulant. Data were directly exported from the Cytobank platform.

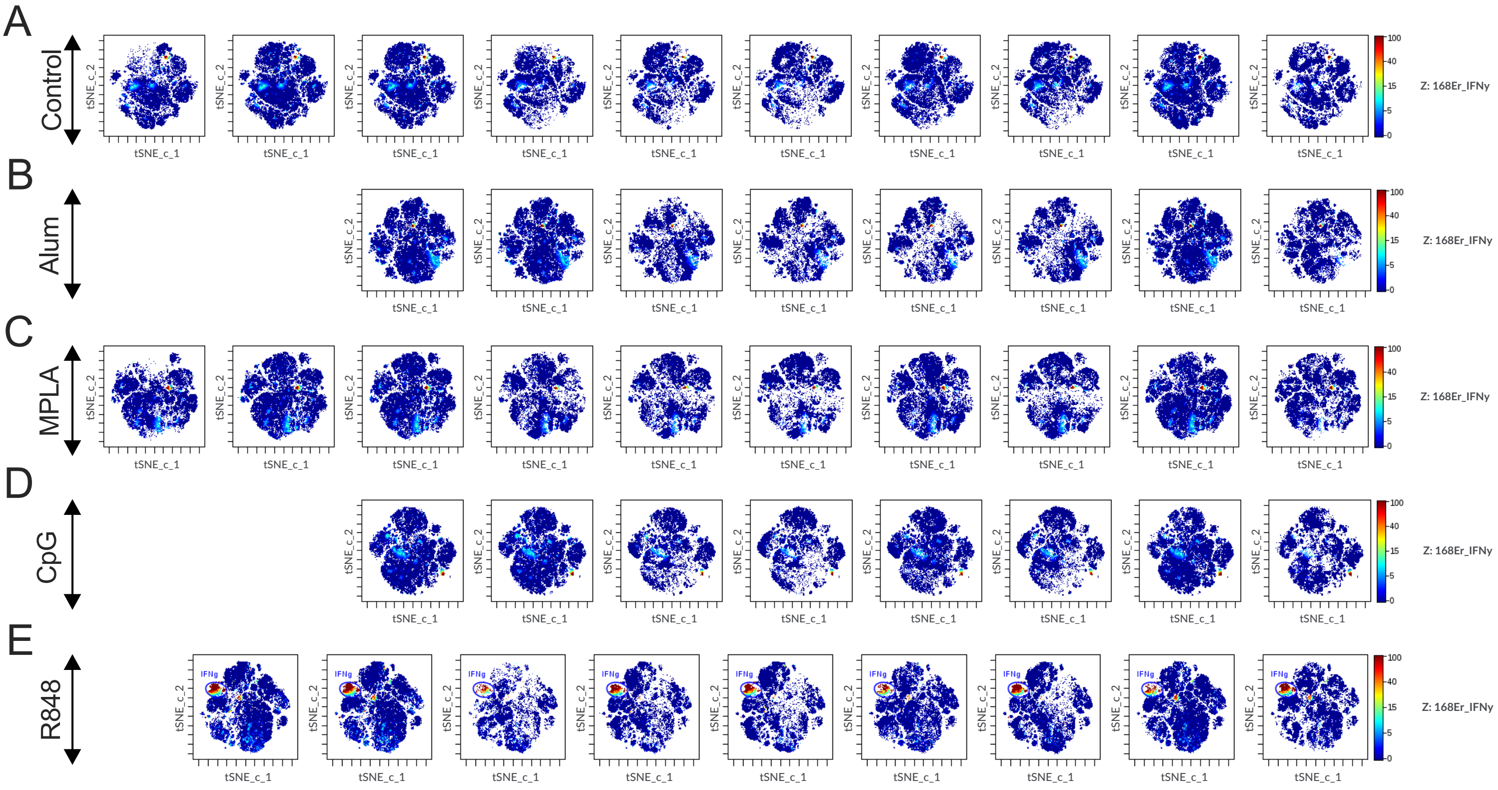

F

% of population for CD45$^+$ CD66a$^-$ IFNγ$^+$ cells of CD45$^+$ CD66a$^-$

Control	1.147143	2.072857	2.074286	0.23	0.21	0.267143	0.245714	0.505714	1.162857	0.277143
Alum			2.047143	2.061429	0.154286	0.228571	0.212857	0.481429	0.934286	0.33
MPLA	1.061429	2.464286	2.228571	0.167143	0.221429	0.23	0.261429	0.487143	1.325714	0.495714
CpG			3.341429	3.455714	0.344286	0.324286	0.288571	0.468571	1.111429	0.662857
R848		7.711429	6.104286	1.572857	2.771429	3.712857	1.101429	3.625714	2.261429	5.288571

Supplementary Figure 6: TLR7/8a (R848) has greater IFNγ inducing efficacy than other PRRa in human cord BMCs. Non-stimulated cord BMCs were treated as (A) control group. BMCs were stimulated with (B) alum (10μg/ml), (C) MPLA (100ng/ml), (D) CpG (5μM) and (E) R848 (5μM) for 18h from the newborn cohort. IFNγ expression (in *Z*-axis channel) overlaid on tSNE-CUDA embedding. Color indicates median metal intensity of IFNγ expression ranging from low (blue) to high (red). 70K events of MNCs from each donor used in visualization with tSNE-CUDA. For R848 stimulation in (E), a manual gate in tSNE-CUDA plot indicates (for visualization, not for quantification) the predominant island expressing IFNγ in MNCs. (F) Proportion (frequencies) of IFNγ$^+$ cells in MNCs are shown for each stimulation. The FCS files are excluded from the tSNE-CUDA analysis which does not possess 70K events of MNC population during equal event sampling as described in the Methods section. Data were directly exported from the Cytobank platform.

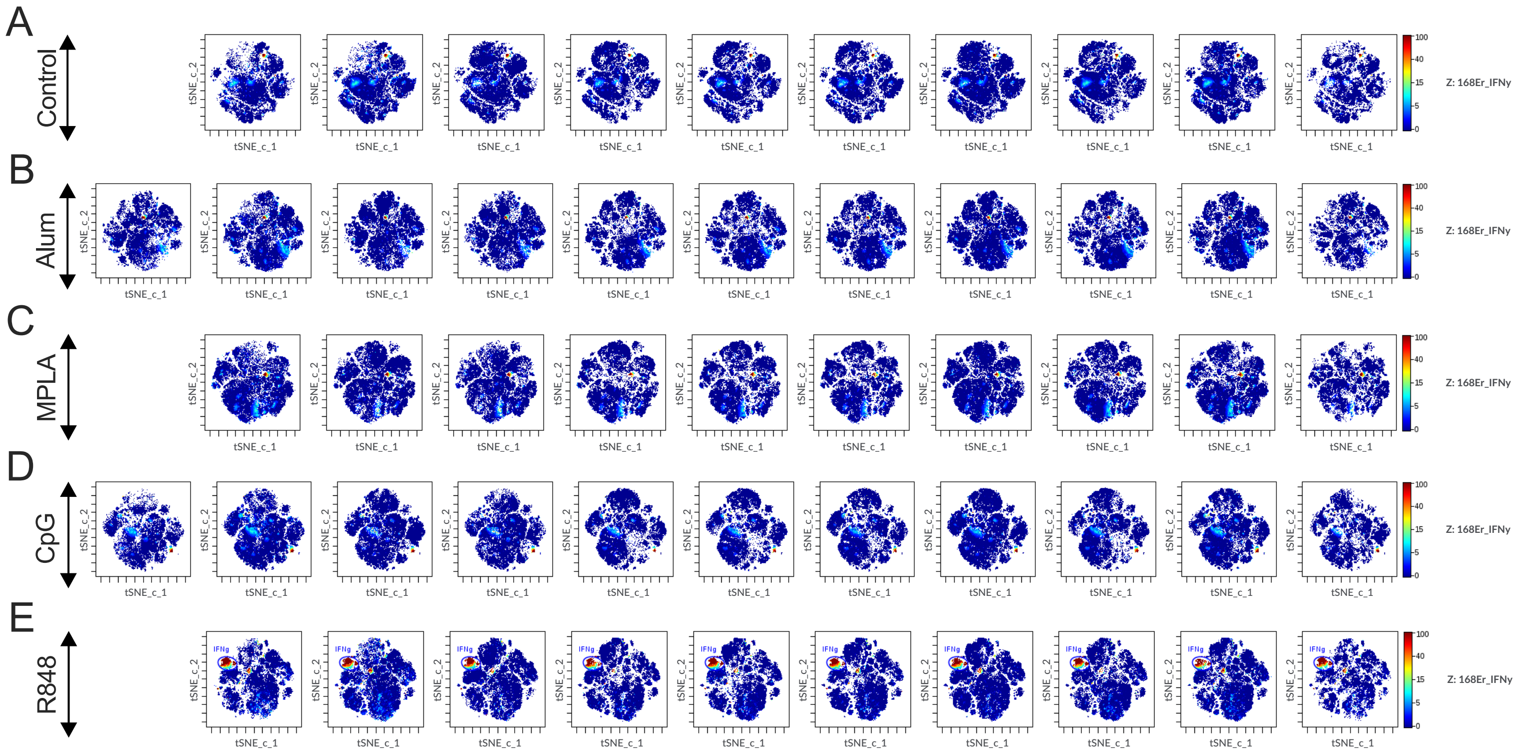

F

% of population for CD45$^+$ CD66a$^-$ IFNγ$^+$ cells of CD45$^+$ CD66a$^-$

Control		1.208571	4.228571	0.47	0.3	0.3	0.235714	0.317143	0.724286	1.442857	0.34
Alum	1.347143	3.671429	0.384286	0.751429	0.335714	0.295714	0.252857	0.281429	0.688571	1.314286	0.365714
MPLA		3.377143	0.428571	0.75	0.291429	0.402857	0.175714	0.302857	0.871429	1.5	2.811429
CpG	1.391429	4.27285714	0.44571429	0.82	0.3	0.34	0.271429	0.7	0.878571	1.527143	0.572857
R848		8.02142857	6.5	5.238571	2.724286	4.651429	3.098571	2.694286	4.315714	3.258571	11.79

Supplementary Figure 7: TLR7/8a (R848) has greater IFNγ inducing efficacy than other PRRa in human adult BMCs. Non-stimulated adult BMCs were treated as (A) control group. BMCs were stimulated with (B) alum (10µg/ml), (C) MPLA (100ng/ml), (D) CpG (5µM) and (E) R848 (5µM) for 18h from the adult cohort. IFNγ expression (in Z-axis channel) overlaid on tSNE-CUDA embedding. Color indicates median metal intensity of IFNγ expression ranging from low (blue) to high (red). 70K events of MNCs from each donor used in visualization with tSNE-CUDA. For R848 stimulation in (E), a manual gate in tSNE-CUDA plot indicates (for visualization, not for quantification) the predominant island expressing IFNγ in MNCs. (F) Proportion (frequencies) of IFNγ$^+$ cells in MNCs are shown for each stimulation. The FCS files are excluded from the tSNE-CUDA analysis which does not possess 70K events of MNC population during equal event sampling as described in the Methods section. Data were directly exported from the Cytobank platform.

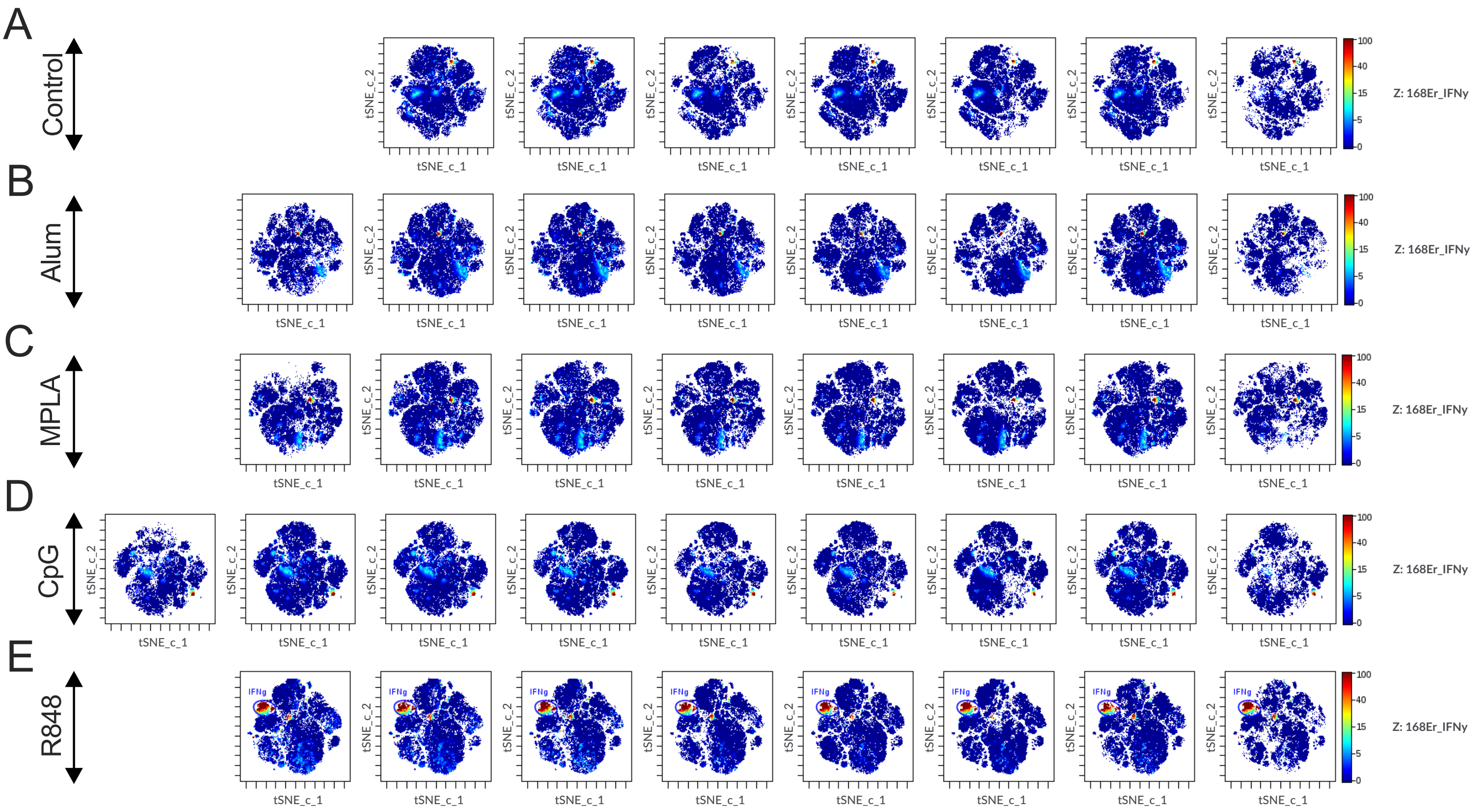

F

% of population for $CD45^{+}$ $CD66a^{-}$ $IFN\gamma^{+}$ cells of $CD45^{+}$ $CD66a^{-}$

Control			3.517143	3.388571	0.697143	0.315714	0.51	1.262857	0.318571
Alum		0.805714	3.407143	3.712857	0.768571	0.314286	0.422857	1.114286	0.395714
MPLA		0.88	2.451429	3.648571	1.302857	0.298571	0.447143	1.225714	0.707143
CpG	1.78857143	5.10285714	3.695714	3.097143	0.522857	0.404286	0.724286	1.234286	0.444286
R848		7.424286	7.16	5.838571	5.17	2.254286	4.58	3.251429	6.034286

Supplementary Figure 8: TLR7/8a (R848) has greater IFNγ inducing efficacy than other PRRa in human elder BMCs. Non-stimulated elder BMCs were treated as (A) control group. BMCs were stimulated with (B) alum (10µg/ml), (C) MPLA (100ng/ml), (D) CpG (5µM) and (E) R848 (5µM) for 18h from the elder cohort. IFNγ expression (in Z-axis channel) overlaid on tSNE-CUDA embedding. Color indicates median metal intensity of IFNγ expression ranging from low (blue) to high (red). 70K events of MNCs from each donor used in visualization with tSNE-CUDA. For R848 stimulation in (E), a manual gate in tSNE-CUDA plot indicates (for visualization, not for quantification) the predominant island expressing IFNγ in MNCs. (F) Proportion (frequencies) of $IFN\gamma^{+}$ cells in MNCs are shown for each stimulation. The FCS files are excluded from the tSNE-CUDA analysis which does not possess 70K events of MNC population during equal event sampling as described in the Methods section. Data were directly exported from the Cytobank platform.

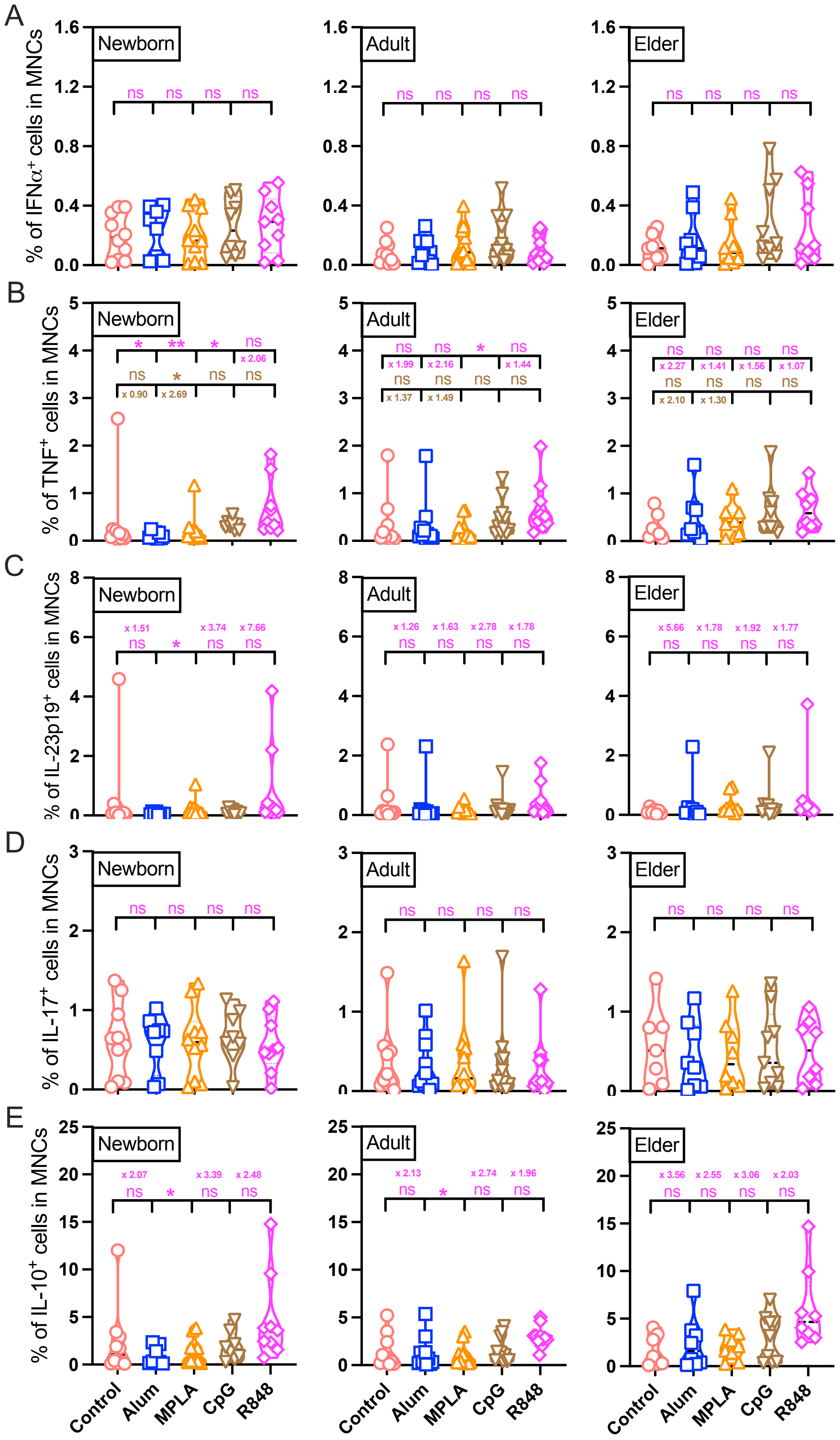

Supplementary Figure 9: Intracellular cytokines profile of MNCs after PRRa stimulation for 18h. BMCs were stimulated with alum (10µg/ml), MPLA (100ng/ml), CpG (5µM) and R848 (5µM) from newborn, adult and elder cohort. Proportion (frequencies) of (A) IFNα^{+}, (B) TNF^{+}, (C) IL-23p19^{+}, (D) IL-17^{+} and (E) IL-10^{+} cells in MNCs are shown for each stimulation. Mean fold differences between R848 or CpG and rest PRRa adjuvants are also shown. Statistical comparison was performed using either one-way ANOVA or nonparametric Kruskal-Wallis test corrected for multiple comparisons; *$p < 0.05$, **$p < 0.01$, ns denoted non-significant. Each dot represents a single participant (n=7-11 per group).

A

Newborn

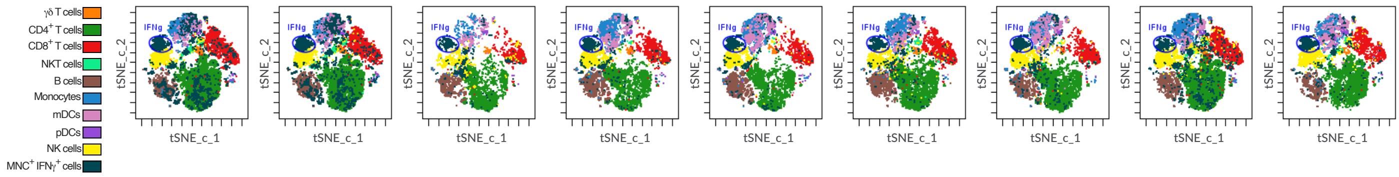

γδ T cells	0.371	0.304	0	0.155	0.077	1.686	0.0788	1.642	0.108
CD4+ T cells	17.544	14.978	11.989	2.784	2.424	6.615	5.043	33.418	3.052
CD8+ T cells	4.428	5.429	4.36	1.237	1	2.983	1.064	13.392	5.159
NKT cells	1.945	2.176	1.181	0.464	0.731	1.038	0.315	4.359	2.35
B cells	10.578	9.338	1.998	0.464	0.346	1.427	0.709	0.821	0.702
Monocytes	2.242	3.838	1.998	3.247	1.885	16.472	4.413	2.843	1.675
mDCs	0.371	0.702	1.181	0.876	0.616	5.707	1.576	0.379	0.513
pDCs	0.0371	0.304	0.0908	0.0515	0.0385	0	0.118	0.126	0
NK cells	29.622	20.173	29.882	47.68	62.716	25.292	45.863	16.235	45.597
MNC+ IFNγ+ cells	100	100	100	100	100	100	100	100	100

B

Adult

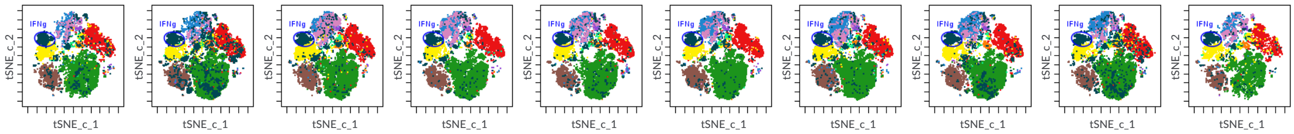

γδ T cells	2.618	2.264	7.281	3.461	6.419	5.486	7.476	0.993	4.472	0.158
CD4+ T cells	3.722	15.604	3	4.248	5.16	4.38	2.545	11.917	19.246	1.963
CD8+ T cells	3.865	20.044	2.672	4.51	2.611	3.734	7.37	2.052	20.956	4.653
NKT cells	1.496	4.901	0.355	1.416	1.29	6.593	3.022	0.397	7.847	1.757
B cells	0.57	2.418	0.491	0.42	0.829	0.922	1.75	0.563	1.052	0.557
Monocytes	1.086	1.648	1.8	4.457	4.822	1.66	2.757	5.594	1.622	0.448
mDCs	0.677	0.615	0.764	1.049	1.351	0.277	1.06	1.225	0.438	0.291
pDCs	0.0534	0.11	0.0545	0	0.123	0.0461	0.106	0.0993	0.132	0.0485
NK cells	33.019	16.857	37.197	43.891	38.851	28.124	43.531	24.793	28.759	55.204
MNC+ IFNγ+ cells	100	100	100	100	100	100	100	100	100	100

C

Elder

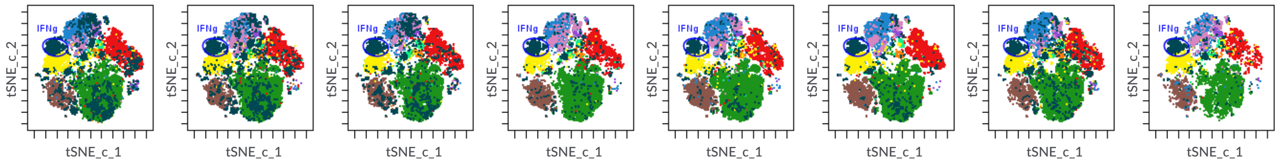

γδ T cells	0.75	0.419	0.367	0.691	1.838	0.717	0.879	0.308
CD4+ T cells	28.42	34.757	17.03	19.342	7.605	7.86	28.427	3.741
CD8+ T cells	6.869	7.741	6.019	14.092	7.985	1.528	11.16	5.634
NKT cells	0.827	1.437	2.08	10.776	3.992	0.499	4.042	2.32
B cells	1.77	1.297	9.494	0.414	1.204	0.374	0.571	0.355
Monocytes	6.388	3.671	4.796	0.995	3.105	6.488	2.153	1.207
mDCs	0.808	1.397	0.832	0.166	0.887	0.499	0.395	0.521
pDCs	0.154	0.0798	0.147	0	0	0.0936	0.351	0.071
NK cells	17.279	12.011	15.439	8.345	35.108	54.71	22.144	44.152
MNC+ IFNγ+ cells	100	100	100	100	100	100	100	100

Supplementary Figure 10: Dissecting TLR7/8a-specific IFNγ producing cell subsets in each study participant. DR analysis using tSNE-CUDA of MNCs from each individual study participant (n=27) after R848 (5µM) stimulation for 18h. Data from 3 participants were excluded for downstream analysis as they did not fit the threshold criteria of 70K events/ individual as described in the Method section. Percent of population varying by overlaid (selected cell populations) of IFNγ^{+} MNCs were shown in tables from (A) newborn, (B) adult and (C) elder cohorts. Data were directly exported from the Cytobank platform.

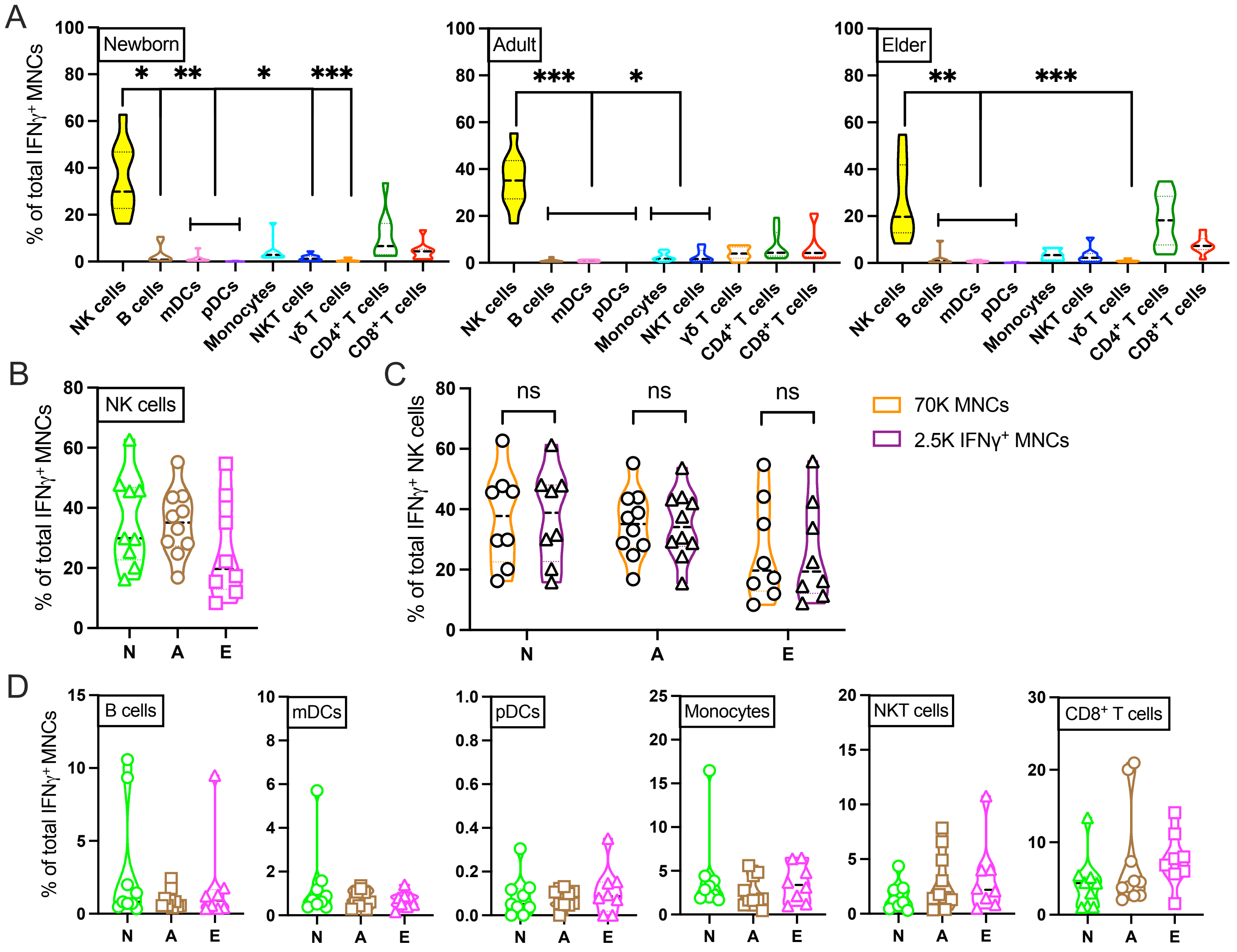

Supplementary Figure 11: Tracking the IFNγ producing cellular lineages. (A) IFNγ production by major immune cell lineages in MNCs after R848 (5µM) stimulation for 18h. (B) Age-specific effect of IFN*γ*+ NK cells in MNC compartment after R848 stimulation. (C) Comparison of the frequency of IFN*γ*+ NK cells in R848 stimulated MNCs by two distinctive tSNE-CUDA runs after selecting 70K MNCs and 2.5K IFN*γ*+ MNCs respectively. Equal sampling option was chosen for each tSNE-CUDA in the Cytobank platform. (D) Frequency of IFN*γ*+ MNCs in B cell, mDC, pDC, monocyte, NKT cell and CD8+ T cell compartment after R848 stimulation. Statistical comparison was performed using either one-way ANOVA or nonparametric Kruskal-Wallis test corrected for multiple comparisons. For S11C, two-tailed paired t-test was used for comparison between 2 groups; *p < 0.05, **p < 0.01, ***p < 0.001, ns denoted non-significant. N stands for newborn; A, adult and E, elder. Each dot represents a single participant (n=7-11 per group).

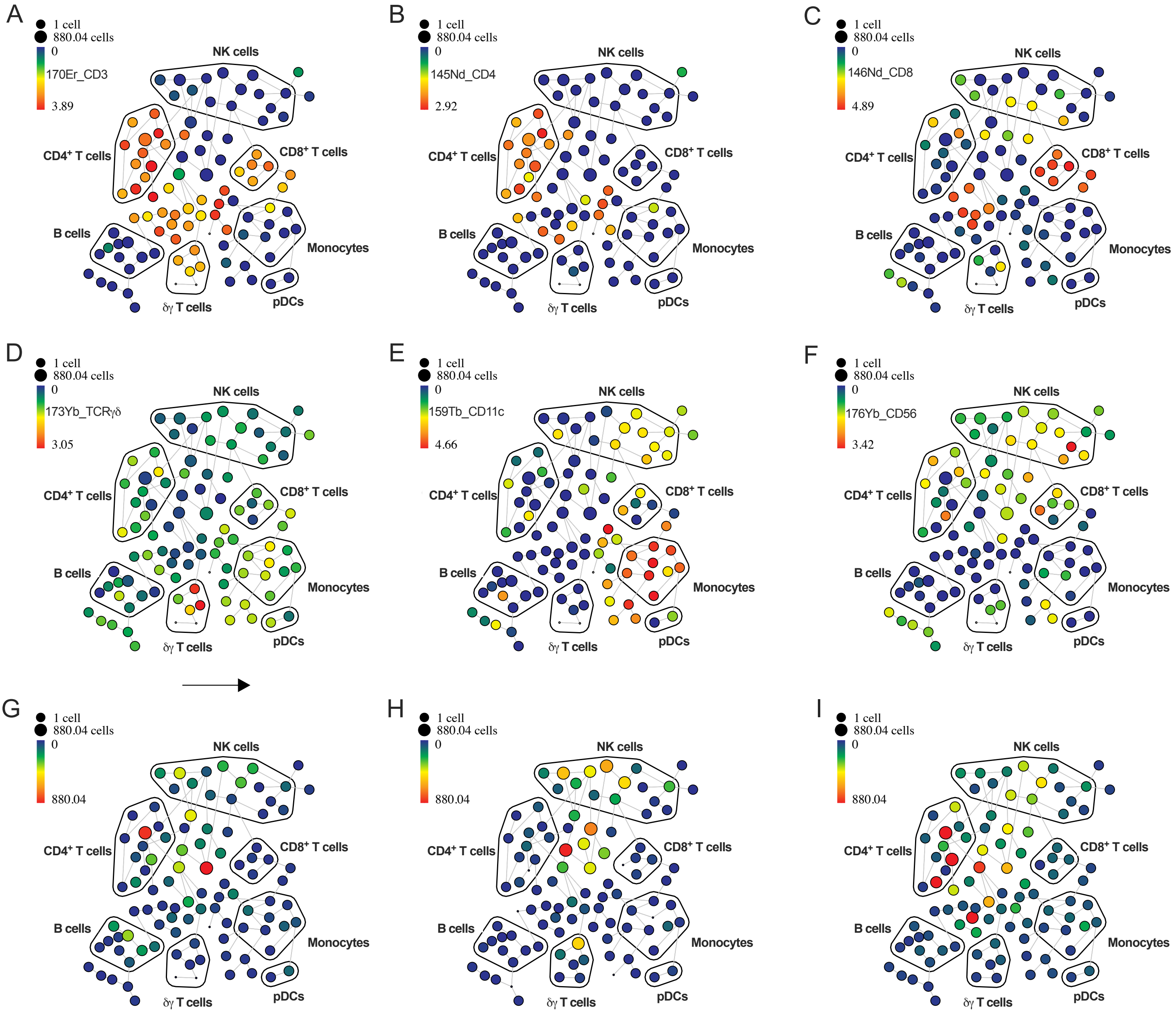

Supplementary Figure 12: Clustering analysis identifies 7 different cellular populations of IFNγ producing cells with age specific differences for γδ T cells in adults and CD4⁺ T cell in later life. Representative SPADE plots using automatic clustering by SPADE algorithm after R848 stimulation. Representative plots from a newborn participant are displaying (A) CD3, (B) CD4, (C) CD8, (D) TCR$\gamma\delta$, (E) CD11c and (F) CD56 expression. Each tree is colored according to the indicated marker. Median metal intensity ranging from low (blue) to high (red) was used to identify 7 different subpopulations described in the text. Cell diversity was portrayed in SPADE trees from representative (G) newborn, (H) adult and (I) elder participants based on cell counts in each node. Each tree (G-I) is colored according to the cell accumulation in each node.

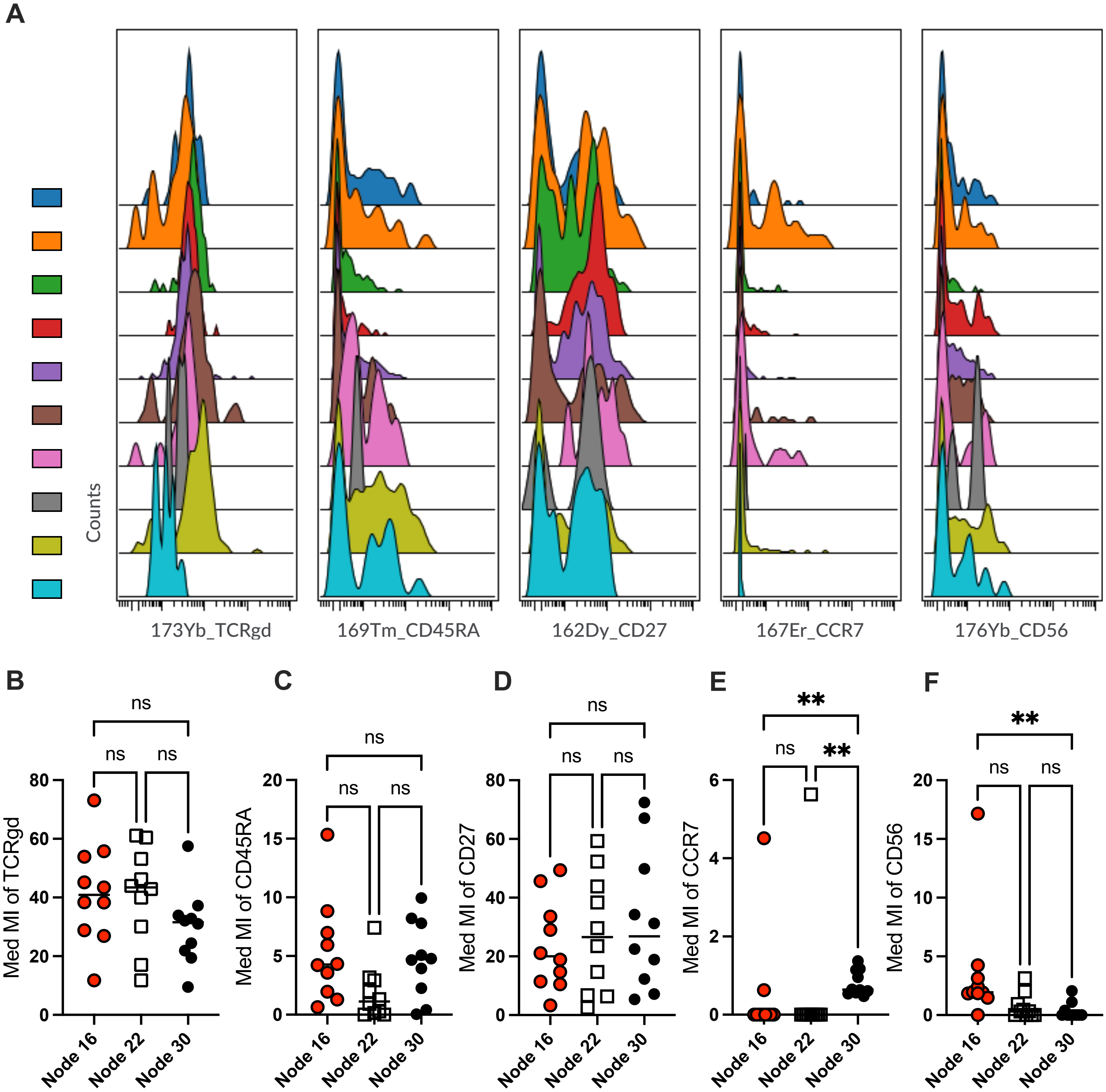

Supplementary Figure 13: Charaterization of nodes (16, 22 & 30) from the SPADE bubble of IFNγ⁺ γδ T cell compartment after TLR7/8a (R848) stimulation.

(A) Representative histograms of node 16 according to the Med MI of (B) TCRgd, (C) CD45RA, (D) CD27, (E) CCR7 and (F) CD56 expression. Data from 10 adult participants were graphed here. Each color (A) and circle (B-F) represent a single participant. Statistical comparison was performed using either one-way ANOVA or nonparametric Kruskal-Wallis test corrected for multiple comparisons; **p < 0.01, ns denoted non-significant.
