# Supplementary material for: Immune profiling of age and adjuvant-specific activation of human blood mononuclear cells in vitro
Source: Commun Biol. 2024 Jun 8;7:709. doi: 10.1038/s42003-024-06390-4 (PMC11162429; doi:10.1038/s42003-024-06390-4)
Supplement: Supplementary file 3 — Description of Additional Supplementary Files [file 42003_2024_6390_MOESM3_ESM.pdf]

Description of Additional Supplementary Files

File name: Supplementary Data 1

Description: Values for all data points in graphs and mean fold changes between groups of interest.

File name: Supplementary Data 2

Description: Participant's identifier with the FCS file names along with independent assay number.
