# Supplementary material for: Immune profiling of age and adjuvant-specific activation of human blood mononuclear cells in vitro
Source: Commun Biol. 2024 Jun 8;7:709. doi: 10.1038/s42003-024-06390-4 (PMC11162429; doi:10.1038/s42003-024-06390-4)
Supplement: Supplementary file 6 — Reporting Summary [file 42003_2024_6390_MOESM6_ESM.pdf]

nature portfolio

Corresponding author(s): Ofer Levy & David J. Dowling

Last updated by author(s): May 24, 2024

Reporting Summary

Nature Portfolio wishes to improve the reproducibility of the work that we publish. This form provides structure for consistency and transparency in reporting. For further information on Nature Portfolio policies, see our Editorial Policies and the Editorial Policy Checklist.

Statistics

For all statistical analyses, confirm that the following items are present in the figure legend, table legend, main text, or Methods section.

n/a	Confirmed	
☐	☒	The exact sample size (*n*) for each experimental group/condition, given as a discrete number and unit of measurement
☐	☒	A statement on whether measurements were taken from distinct samples or whether the same sample was measured repeatedly
☐	☒	The statistical test(s) used AND whether they are one- or two-sided<br>*Only common tests should be described solely by name; describe more complex techniques in the Methods section.*
☒	☐	A description of all covariates tested
☐	☒	A description of any assumptions or corrections, such as tests of normality and adjustment for multiple comparisons
☐	☒	A full description of the statistical parameters including central tendency (e.g. means) or other basic estimates (e.g. regression coefficient) AND variation (e.g. standard deviation) or associated estimates of uncertainty (e.g. confidence intervals)
☐	☒	For null hypothesis testing, the test statistic (e.g. *F*, *t*, *r*) with confidence intervals, effect sizes, degrees of freedom and *P* value noted<br>*Give P values as exact values whenever suitable.*
☒	☐	For Bayesian analysis, information on the choice of priors and Markov chain Monte Carlo settings
☐	☒	For hierarchical and complex designs, identification of the appropriate level for tests and full reporting of outcomes
☒	☐	Estimates of effect sizes (e.g. Cohen's *d*, Pearson's *r*), indicating how they were calculated

Our web collection on statistics for biologists contains articles on many of the points above.

Software and code

Policy information about availability of computer code

Data collection	After acquisition by Helios instrument, samples were normalized and de-barcoded according to manufacturer's recommendation with CyTOF software (version 6.7.1014).
Data analysis	All software and algorithms used in this manuscript have been described in the "Methods" section in detail. A list of all implemented software and algorithms are also included in "Supplementary Table 3".

For manuscripts utilizing custom algorithms or software that are central to the research but not yet described in published literature, software must be made available to editors and reviewers. We strongly encourage code deposition in a community repository (e.g. GitHub). See the Nature Portfolio guidelines for submitting code & software for further information.

Data

Policy information about availability of data

All manuscripts must include a data availability statement. This statement should provide the following information, where applicable:

- Accession codes, unique identifiers, or web links for publicly available datasets
- A description of any restrictions on data availability
- For clinical datasets or third party data, please ensure that the statement adheres to our policy

Data are available in the main article, figures, tables, supplementary materials and in supplementary Excel files, which are available online with this article. Values for all data points in graphs and mean fold changes between groups of interest are reported either in supplementary figures or in the separate Excel file named

"Supplementary Data 1".
Deposited raw quality controlled and assured CyTOF data (FCS files) can be accessed via a registered account without any subscriptions, through the Cytobank Premium platform (https://premium.cytobank.org/cytobank/projects/3668) via the following links.
FCS files for Fig. 1 (https://premium.cytobank.org/cytobank/experiments/474341), Alum stimulation (https://premium.cytobank.org/cytobank/experiments/474346); MPLA stimulation (https://premium.cytobank.org/cytobank/experiments/474666); CpG stimulation (https://premium.cytobank.org/cytobank/experiments/474672) and R848 stimulation (https://premium.cytobank.org/cytobank/experiments/472809) related to Fig. 2-7. Participant's identifier with the FCS file names along with independent assay number are provided in the Supplementary Excel file named "Supplementary Data 2". The same Excel file is also available in the "Attachments" section (in the Cytobank platform) under each stimulation.
For any further information, please email the corresponding author Dr. David Dowling at david.dowling@childrens.harvard.edu or our PVP CyTOF team at pvp.cytof@childrens.harvard.edu.

Research involving human participants, their data, or biological material

Policy information about studies with human participants or human data. See also policy information about sex, gender (identity/presentation), and sexual orientation and race, ethnicity and racism.

Reporting on sex and gender	16 males and 14 females were recruited. No sex-based analysis were performed.
Reporting on race, ethnicity, or other socially relevant groupings	Socially relevant categorization were not performed.
Population characteristics	Peripheral blood was collected from healthy adult (n = 11) and elder volunteers (n = 9), while human newborn cord blood (n = 10) was collected immediately after Cesarean section delivery of the placenta. Births to known HIV-positive mothers were excluded.
Recruitment	Blood samples were collected during the year of 2018.
Ethics oversight	Nonidentifiable human cord blood samples were collected with the approval from the Ethics Committee of the Brigham & Women's Hospital, Boston, MA; Institutional Review Board (IRB) protocol number 2000-P-000117, and Beth Israel Deaconess Medical Center Boston, MA (IRB protocol number 2011P-000118). The requirement for informed consent was waived for the non-identifiable human cord blood samples. Peripheral blood from healthy elder donors was obtained from the outpatient clinics of Brigham & Women's Hospital (Boston, MA) after written informed consent with approval from the IRB (protocol no. 2013P002473). Blood from healthy, adult volunteers was collected after written informed consent with approval from the Ethics Committee of Boston Children's Hospital, Boston, MA (protocol number X07-05-0223). All ethical regulations relevant to human research participants were followed.

Note that full information on the approval of the study protocol must also be provided in the manuscript.

Field-specific reporting

Please select the one below that is the best fit for your research. If you are not sure, read the appropriate sections before making your selection.

☒ Life sciences ☐ Behavioural & social sciences ☐ Ecological, evolutionary & environmental sciences

For a reference copy of the document with all sections, see nature.com/documents/nr-reporting-summary-flat.pdf

Life sciences study design

All studies must disclose on these points even when the disclosure is negative.

Sample size	Sample size for in vitro stimulations were chosen empirically based on the results of previous studies.<br>BMCs from a total of 30 participants (details in "Supplementary Data 2") were prepared for the CyTOF run.
Data exclusions	Data were excluded for downstream analysis if they did not fit the threshold criteria of 70K events (MNC)/ individual as described in the "Methods" section.
Replication	A total of 10 independent assays with BMCs from 30 participants (details in "Supplementary Data 2") matching representatives of newborn, adult and elder samples were prepared to reduce batch effects and each experiment was performed as a single assay.
Randomization	Samples were allocated into independent assays based on matching representatives of newborn, adult and elder participants.
Blinding	Blinding was not performed despite all experiments being carried out in an unbiased manner and statistics were not calculated until the study was complete.

Reporting for specific materials, systems and methods

We require information from authors about some types of materials, experimental systems and methods used in many studies. Here, indicate whether each material, system or method listed is relevant to your study. If you are not sure if a list item applies to your research, read the appropriate section before selecting a response.

Materials & experimental systems

n/a	Involved in the study
☐	☒ Antibodies
☒	☐ Eukaryotic cell lines
☒	☐ Palaeontology and archaeology
☒	☐ Animals and other organisms
☒	☐ Clinical data
☒	☐ Dual use research of concern
☒	☐ Plants

Methods

n/a	Involved in the study
☒	☐ ChIP-seq
☒	☐ Flow cytometry
☒	☐ MRI-based neuroimaging

Antibodies

Antibodies used	We listed the specific information of the metal-tagged antibodies for mass cytometry in the “Supplementary table 1”.
Validation	Commercial antibodies were used according to the manufacturer’s information and this is noted in the manuscript where applicable.

Plants

Seed stocks	Not applicable.
Novel plant genotypes	Not applicable.
Authentication	Not applicable.
